# Brown adipose tissue human biomarkers: Which one fits best? A narrative review

**DOI:** 10.1097/MD.0000000000032181

**Published:** 2022-12-02

**Authors:** Angelo Alito, Angelo Quartarone, Giulia Leonardi, Adriana Tisano, Antongiulio Bruschetta, Francesca Cucinotta, Demetrio Milardi, Simona Portaro

**Affiliations:** a Department of Biomedical, Dental Sciences and Morphological and Functional Images, University of Messina, Messina, Italy; b IRCCS Centro Neurolesi Bonino-Pulejo, Messina, Italy; c Department of Physical and Rehabilitation Medicine and Sports Medicine, Policlinico “G. Martino”, Messina, Italy; d Department of Clinical and Experimental Medicine, University of Messina, Messina, Italy; e Orthopaedic Institute of Southern Italy “Franco Scalabrino”, Messina, Italy.

**Keywords:** adipose tissue, bat activation, brown adipose tissue, brown fat, cold exposure

## Abstract

Adipose tissue (AT) is an endocrine metabolically dynamic active tissue that plays a central role in the systemic energy balance and metabolic regulation. Brown AT represents approximately 1% of adult human AT, with an energy-burning function that uses fat to create heat. Brown AT activity was measured using 18F-fluorodeoxyglucose positron emission tomography/computed tomography. It has been shown that cold exposure could promote brown AT activation. However, many factors, such as aging and body mass index, may interfere with this activity. Many authors have discussed the role of factors specifically secreted by the AT in response to cold exposure. The aim of this review is to properly understand the effects of cold on AT and biomarkers and their possible application in rehabilitation medicine. A comprehensive literature review was performed to identify published studies regarding biomarkers of cold effects on Brown AT searching the following databases: PubMed, Science Direct, and Web of Science, from 2012 to 2022. After evaluation of the inclusion and exclusion criteria, 9 studies were included in this review. We reported the overall influence of cold exposure on brown AT activity, its related biomarkers, and metabolism, demonstrating that the therapeutic role of cold exposure needs to be better standardized. From our data, it is important to design proper clinical trials because most cold applied protocols lack a common and homogeneous methodology.

## 1. Introduction

Adipose tissue (AT) is an endocrine metabolically dynamic active tissue that plays a central role in systemic energy balance and metabolic regulation.^[1]^ In the past 2 decades, scientific interest has developed in this topic due to concerns about obesity and its metabolic sequelae, and to the recognized role of adipocytes in some homeostatic processes.^[2]^ AT, based on its thermogenic potential, is classified as white AT (WAT), beige, or brown AT (BAT), with different structures and functions, even though they all store lipids as triglycerides^[3,4]^ WAT is the primary storage site for lipids without thermogenic capacity; it is the most represented fat type in human adults, and quickly stores and releases lipids in response to various metabolic conditions.^[3,5]^

BAT represents approximately 1% of adult human AT and is mainly localized in the neck and upper body regions.^[6–8]^ BAT has an energy-burning function that uses fat to create heat (i.e., non-shivering thermogenesis) because of the uncoupling protein 1 (UCP1) expression in the mitochondria.^[3]^ Beige or brite (brown-in-white) AT, which develops from WAT in response to chemical signaling or cold exposure (CE),^[9]^ may exert the same BAT metabolic functions, producing a small UCP1 amount upon prolonged CE or direct adrenergic stimulation in a process known as browning.^[10,11]^ UCP1 is located in the inner mitochondrial membrane and uncouples the mitochondrial proton gradient from ATP production.^[12]^ It has been shown that cold activates sympathetic neurons, which release noradrenaline (NE) activating the β3-adrenergic receptor, which has a key-role on UCP1 activation and mitochondrial thermogenesis.^[13]^ On such a basis, UCP1 represents the classic BAT activity biomarker.^[14]^ BAT activity and browning are influenced by several factors, such as CE^[15,16]^ drugs,^[17]^ altered glucose and lipid metabolism^[18–20]^ and dysthyroidism,^[21]^ which trigger thermogenesis. The mechanisms of action of such BAT activators, bringing to an augmented β-adrenergic and/or UCP1 activity and decreasing existing WAT storage, are increasing lipolysis, with a subsequent free fatty acid release from WAT; and conversion of white adipocytes to thermogenic-active beige adipocytes.^[22]^ Such cold-induced thermogenesis processes are attributable to voluntary (i.e., physical exercise) or involuntary (i.e., shivering) skeletal muscle activity.^[23]^ In fact, the same β-adrenergic activity or UCP1 induction may come from: exercise, which is considered a potential signaling cue to stimulate BAT activity and browning^[24,25]^; and shivering, which starts immediately or several minutes after CE, where all the metabolic energy expended even for a little effort performed is released as heat.^[23,26]^ However, few studies have specifically measured the changes in UCP1 levels before and after CE in humans, which is most likely due to the difficulty in obtaining multiple BAT biopsies.^[27]^ Therefore, increased BAT activity has been more easily measured by 18F-fluorodeoxyglucose positron emission tomography (PET)–computed tomography,^[8,28]^ even though this activation is not observed in all humans.^[29]^ In fact, some authors divided the patients into 2 groups: BAT-positive (with PET measured metabolically active BAT) and BAT-negative (with PET undetectable BAT), even though stratified by homogeneous anthropometric characteristics (i.e., body mass index [BMI] and fat mass).^[30,31]^ In addition, other factors, such as aging and BMI, may interfere with BAT activity.^[32,33]^ Aging is a negative regulator of brown adipocyte development and function regulator.^[32]^ In humans, a reduction in BAT and weakened thermogenesis activation is observed with increasing age, possibly due to a phenotypical switch described as whitening (e.g., brown to white-like AT conversion), accompanied by reduced UCP1 expression and activity.^[34]^ This thermogenic defect could also be linked to mitochondrial mature brown adipocyte dysfunction and reduced proliferative expansion of brown adipogenic progenitor cells.^[35]^ Furthermore, BAT may lose the ability to respond to adrenergic stimulation because of a defect in post-receptor signaling events.^[36]^

Body weight is another BAT activation interfering factor. In fact, people with high BMI, after PET study, seems to present a lower BAT body percentage and a reduced activation of the same after stimuli.^[33]^

Several authors, over the years, have discussed the role of factors specifically secreted by AT in response to CE, apart from UCP1.

The aim of this review is to properly understand the effects of cold on AT and biomarkers and their possible application in rehabilitation medicine.

## 2. Methods

**Figure 1.**
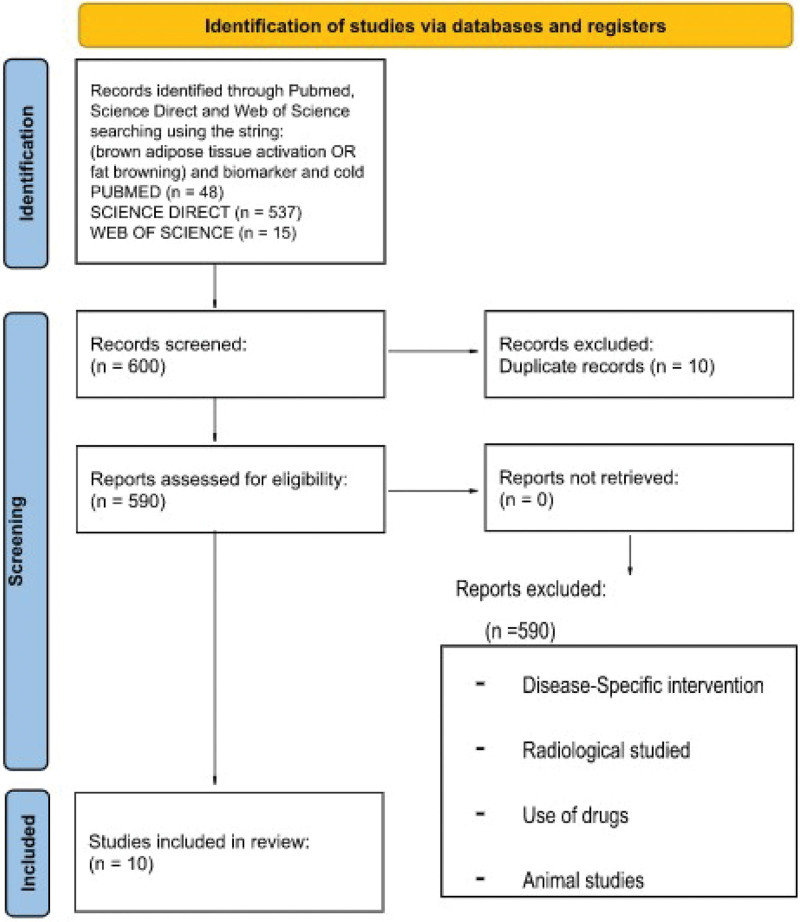
Preferred reporting items flowchart resuming the paper’s selection process.

A comprehensive literature review was performed to identify published studies regarding biomarkers of cold effects on BAT. Two researchers using the same keywords performed the examination process independently. Finally, papers were chosen by consensus. The PubMed, ScienceDirect, and Web of Science databases were searched. The following string was used: (brown adipose tissue activation OR fat browning) AND biomarker AND cold. Identified articles were screened using the following inclusion criteria: study design: randomized controlled trials, review, mini review, articles, written in English, published in indexed journals over the last 10 years (2012–2022), and dealing with brown AT activation biomarkers. Exclusion criteria were drug use, animal studies, radiological studies, and disease-specific interventions. Ethical approval was not required due to the study setting. First, the articles were screened by title and abstract and then by full-text analysis. The following data were collected: study design; cold application modality, biomarkers relevance, and their correlation with brown fat activation. A flowchart of the process is shown in Figure 1. The initial search yielded 600 articles (PubMed, 48; Web of Science, 15; ScienceDirect, 537). Duplicate articles were excluded. After evaluation of the inclusion and exclusion criteria, 9 studies were included in this review.

## 3. Results

We analyzed articles considering BAT activation conditions, including cold exposure and its effects on the human body, considering changes in the blood levels of some biomarkers. The articles included in this review are listed in Table 1. In 2014, Pinho Júnior et al^[37]^ in a clinical trial studied creatine phosphokinase and lactate dehydrogenase after intensive training and a 19 minutes cold-water immersion session. Lactate dehydrogenase levels were lower in the cryotherapy group as the Delta creatine phosphokinase with the finding of lower values. These findings demonstrate a cold effect on muscle damage.

**Table 1:** Synopsis of the studies included in this review.

Author	Year	Study design	Cold application modality	Patients features	Biomarkers	Results	Correlation with BAT activation
Pinho Júnior et al	2014	Clinical Trial with crossover design	Either cold water immersion (5 ± 1°C for 19 min) or no intervention (control) after competition simulation in a crossover modality	10 highly trained males (age 23.3 ± 4.1 yr)	CPK, LDH	For LDH, there was an effect of condition with lower values being found in cryotherapy as compared to control. Delta CPK differed significantly between conditions with lower values being found in cryotherapy as compared to control	Recovery via cold water immersion after simulated competition resulted in less muscle damage
Chen and Pfeifer	2017	Article preview	Exposition whole body	NA	MiRNA (Mir-99b, Mir92a)	BAT, in addition to its capacity to dissipate energy, may regulate metabolism by controlling other organs via exosomal miRNAs	BAT activation reflects on MiRNA increasing that regulates other organs
Villarroya et al	2017	Review	NA	NA	T3, FGF21, neuregulin 4, IGF-1 and IL-6	The secretory properties of brown fat are essential for tissue remodeling adaptations to thermogenic necessities. The endocrine properties of brown adipokines are thought to contribute to the association between BAT activity and a healthy metabolic profile in relation to glucose and lipid homeostasis	Some reports claim that circulating FGF21 or ANGPTL8 levels reflect BAT activity in some conditions but, given the expression and release of these molecules by other tissues, such assertions should be viewed with caution. Blood levels of miRNA-92a have also been proposed to be “negative” biomarkers of BAT activity in humans
Martin et al	2020	Review	NA	NA	UCP1, PGC-1α, Irisin, FGF21	Upregulation of BAT activation markers UCP1, PGC-1α, FGF-21, irisin, natriuretic peptides	Markers of BAT activation increase from both CE and exercise
Soundarrajan et al	2020	Research article	A water-infused suit connected to a temperature control system at a non-shivering temperature for at least 15 min	25 men (18–24 yr) (BMI 19.4–35.9 kg/m^2^)	Fasting glucose, insulin, HbA1c, triglycerides, total cholesterol, LDL, HDL, TSH and fT4, FGF21, IL-6, adiponectin and leptin	Inverse relationship between fasting serum glucose and BAT volume. A marginally significant inverse relationship was also noted between fasting glucose and total BAT activity. No significant correlations were noted for measures of BAT activity or volume and other indicators of adiposity or glucose metabolism	The presence of active BAT may be associated with lower fasting glucose in young men. BAT activity may also be correlated with levels of FGF21, suggesting that BAT may lower glucose levels via an FGF21 dependent pathway
Efremova et al	2020	Research article	Environmental CE	150 Siberian healthy miners living at extremely cold temperatures compared to 29 healthy subjects living in thermoneutral conditions	PBMC expression profile of regulators of BAT activity (CIDEA, PRDM16), white adipocytes browning (HOXC9 and SLC27A1), and fatty acid β-oxidation (CPT1A)	The cold-exposed group showed significantly lower weight, BMI, hip circumference, and PBMC expression of CIDEA, but higher expression of HOXC9 and higher circulating glucose compared to controls	Human PBMC expresses the brown adipocytes marker CIDEA and the browning marker HOXC9, which, varying according to CE, possibly reflect changes in BAT activation and white fat browning
Xiang et al	2020	Randomised, placebo-controlled, double-blinded clinical trial	90 min CE via a temperature-controlled, water-perfused vest and blanket. The water perfusate temperature was adjusted to remain approximately 1°C above the temperature that elicited mild shivering	14 healthy male participants (19–30 yr) (BMI ≤ 25 kg/m^2^)	Fasting glucose, insulin, HbA1c, triglycerides, total, high- and low-density lipoprotein-associated cholesterol and thyroid hormones	A significant increase in total NEFA concentration following CE was positively associated with NE concentration change. Individually, 33 NEFA and 11 oxylipin species increased significantly in response to CE	The concentration of the omega-3 NEFA, DHA and EPA at baseline was significantly associated with BAT activity, and the cold-induced change in 18 NEFA species was significantly associated with BAT activity. Lipid measures were correlated with BAT activity measured via [18F]FDG PET/CT, along with NE concentration (a surrogate marker of sympathetic activity)
Mengel et al	2022	Research article	Water Perfused Blanket connected to a cooling device for 120 min at a non-shivering temperature	170 healthy volunteers divided in BMI groups: NW, OW, OB [male: 83; mean age: 26.0 (23–28)]	Fasting glucose, plasma insulin, leptin, adiponectin, NEFA, TG, CRP, fT3	Fasting glucose levels decreased during CE in all BMI groups. Plasma insulin levels were higher in the OB group compared to the NW and OW groups at basal values and after CE. Serum TG significantly increased during CE in the NW and OW groups, but not in the OB group. Significant increase in plasma NEFA in all groups. Adiponectin serum levels significantly increase during CE in all BMI groups. Significant decrease in plasma leptin concentrations after CE in all groups. Significant increase in serum CRP levels in all BMI groups	The metabolic response to cold is diminished in participants with elevated BMI.
Leow et al	2022	Research article	About 2 h by wearing a cooling vest at a constant temperature of 14.5°C (Cool 58, Polar Products, Ohio). Separated at least 48 h apart, subjects were given capsinoids capsules (12 mg, 8 gel capsules)	NA	BAT secretome, (MTHFD1L)	Exosomal protein, MTHFD1L, to be overexpressed and detectable in plasma for all 3 modes of BAT activation in human subjects, as well as between capsinoids and hyperthyroidism	Plasma concentration of exosomal MTHFD1L correlated with human BAT activity as confirmed by PET-MR

[18F]FDG = 18F-fluorodeoxyglucose, ANGPTL8 = angiopoietin-like protein 8, BAT = brown adipose tissue, BMI = body mass index, CE = cold exposure, CIDEA = cell death inducing DFFA like effector A, CPK = creatine phosphokinase, CPT1A = carnitine palmitoyltransferase 1A, CRP = C-reactive protein, DHA = docosahexaenoic acid, EPA = eicosapentaenoic acid, FGF21 = fibroblast growth factor 21, fT4 = free thyroxine, HbA1c = hemoglobin A1c, HDL = high-density lipoproteins, HOXC9 = homeobox C9, IGF-1 = insulin-like growth factor, IL-6 = interleukin 6, LDH = lactate dehydrogenase, LDL = low-density lipoproteins, MiRNA = microRNAs, MTHFD1L = methylenetetrahydrofolate dehydrogenase (NADP + dependent)1-like, NA = not available, NE = norepinephrine, NEFA = not esterified fatty acids, NW = normal weight group, OB = obese group, OW = overweight group, PBMCP = peripheral blood mononuclear cells, PET/CT = positron emission tomography/computed tomography, PET/MR = positron emission tomography/magnetic resonance, PGC-1α = peroxisome proliferator-activated receptor-gamma coactivator-1α, PRDM16 = PR domain-containing 16, SLC27A1 = solute carrier family 27 member 1, T3 = triiodothyronine, TG = triglycerides, TSH = thyroid-stimulating hormone, UCP1 = uncoupling protein.

In their article, Chen and Pfeifer^[38]^ analyzed BAT activation via cold whole-body exposure, showing an increase in miRNA. In particular, miR-92a expression is inversely correlated with BAT activity. Villaroya et al, in a review, claimed that circulating fibroblast growth factor 21 (FGF21) levels reflect BAT activity under some conditions, but other tissues also express and release these molecules. In addition, they considered miRNA-92a blood concentration as a negative BAT activity biomarker.

In their review, Martin et al^[39]^ compared BAT activation due to cold with that due to exercise trying to parallel these 2 conditions mediated by activation of the sympathetic nervous system. The analyzed biomarkers were UCP1, Peroxisome proliferator-activated receptor gamma coactivator 1-alpha, irisin and FGF21. They found that BAT activation biomarkers were upregulated under both conditions.

Soundarrajan et al,^[40]^ after a non-shivering cold exposition, tested fasting glucose, insulin, glycated hemoglobin HbA1c, triglycerides, total cholesterol, low-density lipoproteins, high-density lipoproteins, thyroid-stimulating hormone and free thyroxine, FGF21, interleukin 6, adiponectin and leptin blood levels. They suggested that active BAT may be associated with lower fasting glucose and FGF 21 levels.

Efremova et al^[41]^ investigated the peripheral blood mononuclear cell (PBMC) expression profiles of regulators of BAT activity (CIDEA, PRDM16), white adipocyte browning (HOXC9 and SLC27A1), and fatty acid β-oxidation (CPT1A) in an extremely cold-exposed environment compared to a thermoneutral-exposed population. Human PBMC express the brown adipocyte marker CIDEA and browning marker HOXC9, suggesting fat browning and BAT activation.

Xiang et al^[42]^ explored the effects of cold on lipid and glucose metabolism, thyroid function, and blood NE concentration. The increase in non-esterified fatty acids concentration after CE was correlated with BAT activity and NE modifications.

In their research article, Mengel et al^[43]^ investigated the variation in fasting glucose, plasma insulin, leptin, adiponectin, non-esterified fatty acids, triglycerides, C-reactive protein, and free triiodothyronine after a device-mediated 120 minutes cooling session at a non-shivering temperature. They found that fasting glucose levels decreased and triglycerides and adiponectin levels significantly increased during cold exposure. After CE, plasma leptin levels were lower and serum C-reactive protein levels were higher than before cold exposure. However, the response to cold was diminished in participants with an elevated BMI.

Leow et al^[31]^ showed that plasma exosomal methylene tetrahydrofolate dehydrogenase (NADP + dependent) 1-like overexpression correlates with human BAT activity, as confirmed by PET–magnetic resonance imaging. This increased level was observed for all 3 BAT activation modes analyzed (cold exposure, capsinoid intake, and hyperthyroidism).

## 4. Discussion

To date, CE is often applied with the aim of reducing local metabolism and inflammatory responses; however, there is insufficient supporting evidence for such an effect in humans. The primary effect of CE is to maintain a reduction in intramuscular temperature for as long as possible, particularly in the immediate stages following injuries, intense exercise, or other clinical conditions, to delay the proliferation of secondary damage.^[44]^

This review reports the overall influence of CE on BAT activity, its related biomarkers, and metabolism, demonstrating that the therapeutic role of CE needs to be better standardized. In fact, CE has been shown to induce various metabolic adjustments, leading to an increase in BAT activation and metabolic heat production; however, several factors may interfere with such processes, depending on the timing and duration of CE, chronic inflammation states, metabolic diseases, dysthyroidism, and patients’ anthropomorphic features, including individual response to BAT activation (Sun, 2018).

Several studies have demonstrated that the upregulation of UCP1 expression plays a role in BAT activation, dissipating heat from free fatty acid, and decreasing body temperature during CE.^[39,45]^ As Efremova and colleagues showed, there are other potential BAT-activity markers, such as brown adipocyte marker CIDEA and beige adipocyte marker HOCX9, which are expressed in PBMC in a cold-environment-exposed human cohort. During BAT activation, lipolytic conditions are generated, leading to a reduction in CIDEA levels because of its role in liposynthesis, which inhibits UCP1 expression.^[46]^ Conversely, they found increased HOXC9 expression, showing WAT-to-beige conversion after CE.^[41]^ Several lines of evidence suggest that AT lipid metabolism-secreted products may contribute essential regulatory cues to integrate nutrient handling in ATs and other organs, thereby affecting systemic lipid homeostasis and development of metabolic dysfunction development.^[47,48]^ For instance, cardiolipins in BAT were recently described as important regulators of energy metabolism,^[49]^ and the thermogenic lipokine 12,13-dihydroxy-9Z-octadecenoic acid is involved in BAT activation in response to cold, leading to an increase in fatty acid (FA) uptake.^[50]^ This is important because excess serum FA levels may trigger inflammatory responses by activating toll-like receptors, which could lead to insulin resistance.^[51]^ In dysfunctional AT, an increased appearance of lipotoxic intermediates such as ceramides and diacylglycerol have been observed.^[52]^ Diacylglycerols can activate certain protein kinase C isoforms that inhibit insulin signaling in the liver and muscles.^[53]^ Additionally, FAs are ceramide biosynthesis precursors that have been linked to oxidative stress, lipotoxicity, and insulin signaling inhibition.^[54]^ The inhibition of ceramide synthases prevents their formation, promotes WAT browning, and improves glucose and lipid metabolism.^[55]^

In line with this evidence, Soundarrajan et al^[40]^ supported the CE BAT activation effects on glucose metabolism. This effect may be related to increased circulating levels of FGF21 because its cold-induced overexpression augments lipid cell glucose intake.^[56]^

Other promising markers include exosomes,^[38]^ which are circulating small lipid vesicles carrying proteins and nucleic acids, including microRNAs (miRNAs) and methylene tetrahydrofolate dehydrogenase (NADP + dependent) 1-like, which play an essential role in cellular communication between cells.^[31]^ Exosomal miRNAs function as signaling molecules that regulate the transcription of their target genes and can cause the phenotypic transformation of recipient cells.^[57]^ Moreover, it has been shown that miRNA levels in BAT-derived exosomes change after BAT activation in vitro and in vivo. Thus, BAT-derived exosomes could be used as potential biomarkers of BAT activity as a valid alternative and noninvasive technique compared to 18F-fluorodeoxyglucose PET-computed tomography.^[31]^ Thus, a better understanding of BAT-derived exosomes and their role in metabolism could be a good strategy to improve metabolic crosstalk with other organs and biomarkers, consequently increasing BAT activity.^[58]^

All of these aspects are worth mentioning because their effects on functional recovery from injury or muscle damage need to be better clarified, and the current best available evidence is often misunderstood or misconstrued. This topic has faced several limitations over the years, as demonstrated by this review, because of the heterogeneity among the applied CE protocols and considered outcome measures.

From such data, it is important to design proper clinical trials, as most of the applied protocols lack a common and homogeneous methodology. It would be interesting to study the effects of different temperatures more precisely (is there a dose-effect?), duration and number of exposures, patients’ features (i.e., anthropometric characteristics, gender, associated diseases, age), application sites (local or whole body) with the same CE device, outcome measures considered, and wide-scale applicability.

In conclusion, this review will hopefully stimulate professionals to set large-scale clinical trials, possibly divided on specific diseases, to define specific guidelines and best suited protocols to optimize and improve patients’ health.

It is quite possible that CE’s potential benefits of CE have been limited by the short duration of application and undosed CE modalities (i.e., ice therapy or ice bath).

Future studies should be carried out on the real existence of a reliable, cheap, and easily applicable molecule to confirm BAT activation and its consequent effects on other organs.

## Acknowledgments

The publication was created with the co-financing of the European Union—FSE-REACT-EU, PON Research and Innovation 2014-2020 DM 1062/2021.

## Author contributions

**Conceptualization:** Angelo Alito, Simona Portaro.

**Data curation:** Demetrio Milardi.

**Funding acquisition:** Angelo Quartarone.

**Investigation:** Giulia Leonardi.

**Methodology:** Adriana Tisano, Demetrio Milardi.

**Supervision:** Francesca Cucinotta.

**Validation:** Angelo Quartarone.

**Visualization:** Antongiulio Bruschetta, Francesca Cucinotta.

**Writing – original draft:** Angelo Alito, Giulia Leonardi.

**Writing – review & editing:** Simona Portaro.
